# What do medical students know about e-cigarettes? A cross-sectional survey from one U.S. medical school

**DOI:** 10.1186/s12909-018-1134-1

**Published:** 2018-03-02

**Authors:** Katie Hinderaker, David V. Power, Sharon Allen, Ellen Parker, Kolawole Okuyemi

**Affiliations:** 1 University of Minnesota - St Joseph’s Family Medicine Residency Program, 580 Rice Street, St. Paul, MN 55103 USA; 2 Department of Family Medicine and Community Health, University of Minnesota, Minneapolis, USA; 3 Sanford Health, Fargo, ND USA; 4 Department of Family & Preventive Medicine, University of Utah, Salt Lake City, USA

**Keywords:** E-cigarettes, Alternative nicotine products, Electronic cigarette, Medical student education, Medical school curriculum development

## Abstract

**Background:**

Although electronic cigarette (e-cigarette) use has rapidly increased, there is little data about what United States medical students know or are taught about them. This study examined medical students’ experiences, knowledge, and attitudes regarding e-cigarettes, as well as their evaluation of their education on e-cigarettes.

**Methods:**

A cross-sectional online survey of medical students currently enrolled at the University of Minnesota Medical School (*n* = 984) was conducted over a three-week period in August and September 2015. Primary outcomes included students’ personal experiences with e-cigarettes, knowledge and attitudes about e-cigarettes, and students’ assessment of their education on e-cigarettes.

**Results:**

66.9% medical students completed the survey. 58% (*n* = 382) of participants identified as female. 35.8% (*n* = 235) were “not sure” whether e-cigarettes were approved by the FDA for smoking cessation, while 4.1% (*n* = 27) falsely believed they were. While 82.9% (*n* = 543) agreed or strongly agreed that they felt confident in their ability to discuss traditional cigarette use with patients, only 12.4% (*n* = 81) agreed or strongly agreed that they felt confident in their ability to discuss e-cigarettes with patients. 94.8% (*n* = 619) of participants believed that they had not received adequate education about e-cigarettes in medical school. A higher proportion of males reported ever using an e-cigarette.

**Conclusions:**

The gaps in medical student knowledge and wide variances in attitudes about e-cigarettes at one medical school together with their report of inadequate education in an environment of increasing use of e-cigarette use in the U.S. speaks to a need for the development of medical school curriculum on e-cigarettes.

**Electronic supplementary material:**

The online version of this article (10.1186/s12909-018-1134-1) contains supplementary material, which is available to authorized users.

## Background

In recent years, there has been a dramatic increase in the use of electronic cigarettes (e-cigarettes) among the U.S. population. One large U.S. survey found up to a six-fold increase in the prevalence of adults reporting ever use of e-cigarettes from 2010 to 2013 alone [1]. Additionally, the number of adults reporting current use of e-cigarettes has more than doubled in the same time period [1, 2]. The American Medical Association, American Heart Association, and American Lung Association advise physicians to discuss e-cigarette use with patients and suggest alternative strategies for smoking cessation [3–5]. At the time of this study, the FDA had not issued any statement concerning e-cigarettes; however a rule finalized in 2016 will enable the FDA to regulate e-cigarettes in the same way that it regulates cigarettes and other tobacco products [6].

Medical students’ perspectives on e-cigarettes are likely unique and may be important for future smoking cessation efforts. Ever use of e-cigarettes is highest under age 25: 21.6% of adults ages 18–24 have ever tried an e-cigarette compared to 12.6% of the overall adult population [7]. Therefore, given their youth, medical students may have had greater contact with peer e-cigarette users than practicing physicians. However, they may be less likely than their peers to be personal users due to their health specific education.

There is limited data about medical student attitudes toward e-cigarettes. A small 2014 survey of 80 medical students in Spain revealed that 41% of participants thought e-cigarettes were “safe” and 15% thought they should be allowed in public spaces [8]. To date, there is only one published survey of U.S. medical students’ attitudes toward alternative tobacco products including e-cigarettes from New York University School of Medicine in 2014 [9]. This study found that 4.1% of participants had ever tried e-cigarettes while 1.6% were current users. Respondents also reported feeling less confident providing counseling regarding alternative tobacco products than cigarette cessation counseling.

Our overarching goal was to define gaps in medical student education regarding e-cigarettes that could better inform curricular development for the education of future physicians about e-cigarette use. The aim of this study was to assess medical students’ knowledge, attitudes and experience with e-cigarettes as well as self-reported sources of information both in and outside of the medical school curriculum. We hypothesized that medical students would have little knowledge of e-cigarettes and would report limited exposure to instruction on e-cigarettes in the medical school curriculum. We also hypothesized that medical students would report low personal e-cigarette use but would report higher levels of personal acquaintances using e-cigarettes.

## Methods

### Participants and setting

All current registered medical students at the University of Minnesota (*n* = 984) were sent an online link to a survey via class email listservs. The survey was open from 8/30/2015 to 9/18/2015. The link was re-sent weekly until study completion. The same survey link was also distributed on class Facebook groups restricted to University of Minnesota medical students. One week prior to survey closure, in-person announcements were made to first and second year classes to encourage participation by non-responders. Caribou coffee shop e-gift cards ($5) were offered and provided to the first 800 participants. This study was granted an Exempt status by the University of Minnesota Institutional Review Board (study number 1506E74542) and received approval from the University of Minnesota Medical School student council.

### Measures

Survey items were developed based on prior surveys of U.S. physicians regarding e-cigarettes [10–13] and a survey of medical students in Spain [8].The entire survey, including demographics, was collected anonymously. All participants were asked at least 17 of 29 questions (completion of the remainder depended on prior survey question responses) and the survey was designed to take 5 min. Respondents had to enter their individual school email address after completing the survey to receive their $5 gift card, ensuring minimal duplicate responses. Email addresses were kept separate from surveys to preserve the anonymity of respondents.

Survey questions were divided into three categories: Experience, Knowledge and Attitudes, and Education. The full survey is available in an additional file (see Additional file 1). Participants were asked if they had ever personally used an e-cigarette, if they were currently using e-cigarettes, and if they had family members or close friends who used e-cigarettes. As e-cigarettes are a relatively new technology with minimal long-term data and conflicting data regarding safety and efficacy in smoking cessation, participants were asked if they knew whether e-cigarettes were FDA approved for smoking cessation (which they were not at the time of this study) to further assess knowledge regarding e-cigarettes using a question with an absolute correct response. Participants did not have to complete every question to complete the survey. All question responses were considered in data analysis whether all questions were answered or not.

### Analyses

Study data were collected and managed using REDCap electronic data capture tools [14]. Our primary outcomes were: students’ personal experiences with e-cigarettes, knowledge about e-cigarettes, and students’ assessment of their education on e-cigarettes. Secondary analyses were performed to determine if other variables were associated with differences in responses regarding knowledge and attitudes about e-cigarettes. For these analyses, the independent variables included gender, race, ethnicity, age, year in medical school, or having ever used an e-cigarette. Dependent variables included correct vs incorrect response (FDA approval), yes vs no to questions regarding attitudes toward e-cigarettes, and levels of agreement with statements about e-cigarettes. For the question “Are e-cigarettes approved by the FDA for smoking cessation?”, a binary analysis was performed between two groups: those who responded correctly “no” versus the incorrect responses “yes” and “not sure” in another. For other questions with the possible responses of “yes”, “no”, and “not sure”, the “yes” and “no” responses were compared using logistic regression with a stepwise procedure for model selection while “not sure” responses were excluded from further analysis. For the statements where participants identified their level of agreement or disagreement on a five-point Likert scale, a proportional odds model with stepwise model selection was used. Fisher’s exact test was used to compare gender, ethnicity, and race between the participants who had ever tried e-cigarettes and the rest of the sample. Question responses regarding personal experiences, knowledge, and assessment of education about e-cigarettes from each of the medical school classes (years 1–4) were compared pairs-wise with each other class year. For the comparisons between class years, Tukey’s method was used to correct for multiple analyses. *P* values < 0.05 were considered statistically significant. All surveys including at least one response were analyzed. Data analysis was performed using R software program (version 3.1.3).

## Results

### Participants

The response rate was 66.9% (658/984). Of the 658, 643 participants completed all survey questions asked of them (65.34%). Results reported for each question are of total respondents to that question. Demographics of respondents are shown in Table 1.14.7% of participants (*n* = 97/658) had ever tried an e-cigarette. Four students were identified as current users (0.6% of sample), two using e-cigarettes weekly and two using e-cigarettes daily. 21.6% (*n* = 142/658) reported having immediate family members or close friends who use e-cigarettes.

**Table 1:** Demographics of Survey Respondents

	N^a^ = 659
Age, mean ± SE	25.4 ± 2.8
Gender, n (%)	
Female	382 (58.0%)
Male	276 (41.9%)
Other	1 (0.2%)
Race and Ethnicity, *missing n = 3*	
White	502 (76.5%)
Asian	77 (11.7%)
Black/African American	22 (3.4%)
Other/More than one race	55 (8.4%)
Hispanic or Latino	26 (4%)
Medical School Year, *missing n = 1*	
MS1	190 (28.9%)
MS2	181 (27.6%)
MS3	127 (19.3%)
MS4	138 (21.0%)
Special Medical School Program, *missing n = 1*	
M.D./Ph.D.	24 (3.6%)
LIC^b^	20 (3.0%)
Flex MD^c^	11 (1.7%)
E-cigarette Use	
Ever use	97 (14.7%)
Current use	4 (0.6%)

*SE* standard error

^a^includes demographics of one survey non-responder

^b^LIC = Longitudinal Integrated Clerkship

^c^Flex MD = students who elect to extend medical school for an approved purpose (eg. MPH, international experience, etc.)

### Main outcomes

**Fig. 1:**
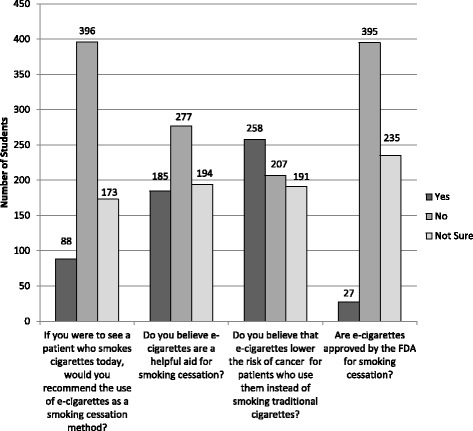
E-Cigarette Knowledge and Attitudes of Year 1–4 Medical Students

**Fig. 2:**
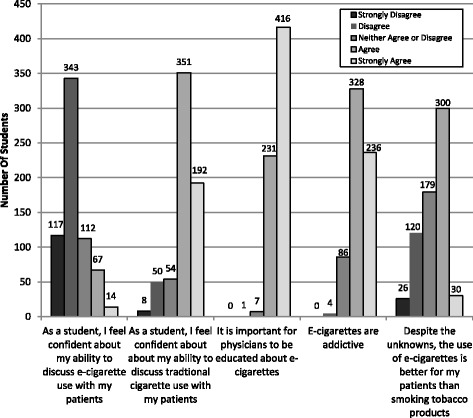
Medical Student Agreement with the Following Statements

**Fig. 3:**
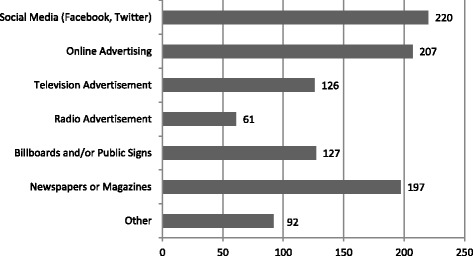
Where have you received information about e-cigarettes outside of medical school?

Participants’ responses regarding knowledge and attitudes about e-cigarettes are shown in Figs. 1 and 2. Regarding education, 84.7% (*n* = 554/654) stated they had not received any education about e-cigarettes in medical school. Of the 15.3% (*n* = 100/654) who reported receiving education about e-cigarettes in medical school, 48% of these (*n* = 48/100) cited a required lecture in years 1 or 2. 30% (*n* = 30/100) had received education through a student interest group or optional lunch lecture, 23% (*n* = 23/100) learned about e-cigarettes through an informal interaction with an attending physician or team, and 10% (n = 10/100) learned through a lecture or discussion in a year 3 or 4 required clerkship. 95% of respondents (*n* = 619/653) did not feel like they had received adequate education about e-cigarettes in medical school. When asked where in the curriculum they thought that education should be included, 65.3% (*n* = 428/655) suggested during a required lecture in years 1 or 2 and 30.1% (*n* = 197/655) suggested it be part of a required clerkship in years 3 or 4. Further, 79.3% (*n* = 142) of the 179 who responded to the open-ended question “Which clerkship?” wrote in “Family Medicine” or a primary care clerkship. Participants’ responses to the question “Have you received any information about e-cigarettes outside of medical school?” are shown in Fig. 3. The most popular answer was through social media such as Facebook or Twitter (*n* = 220/518).

### Secondary outcomes

Students who had ever used an e-cigarette in past were more likely to be male than female (*p* < .001). There were no other statistically significant differences in ever-use of e-cigarettes by age, ethnicity, or race. There were minimal inconsistent differences in question responses by race or ethnicity. Older students were more likely to think e-cigarettes are addictive (*p* < .01) and reported higher confidence discussing traditional cigarette use with patients (*p* < .05) than their younger counterparts. Both male students and students who had ever tried e-cigarettes were more likely to think e-cigarettes have lower risk of causing cancer than traditional cigarettes; think e-cigarettes are helpful for smoking cessation; more likely to recommend e-cigarettes to patients for smoking cessation; agree that the use of e-cigarettes is better for patients than tobacco products; and more likely to feel confident about discussing e-cigarette use with patients than female students and students that had not tried e-cigarettes (*p* < .001 for all responses from both groups), although there was likely confounding between the male group and ever-users due to statistically significant overlap. Ever-users of e-cigarettes were also more likely to think e-cigarettes are addictive than never users (*p* < .01).

As might be expected, MS3s (third-year medical students) and MS4s (fourth-year medical students) were more likely to correctly report that e-cigarettes were not FDA approved for smoking cessation (*p* < 0.001) and report higher confidence discussing traditional cigarettes with patients (*p* < 0.001) than MS1s (first-year medical students). MS3s and MS4s were also more likely to have reported they received education about e-cigarettes (*p* < 0.001) than MS1s and MS2s. However, there were no statistically significant differences by year in school for confidence discussing e-cigarettes or feeling adequately educated about e-cigarettes.

## Discussion

The purpose of this study was to assess the experiences, knowledge, attitudes, and education of medical students toward e-cigarettes with the overarching goal of identifying educational gaps. Regarding experience with e-cigarettes, we discovered an incidence of ever-use of e-cigarettes among medical students in our sample at 15% and a fairly low incidence of current use at less than 1%. A significant number of participants reported family and friends using e-cigarettes. Knowledge and attitudes about e-cigarettes were highly variable among the students in our sample. Over one-third of participants were unsure or incorrect regarding FDA approval of e-cigarettes. There was no clear consensus among participants regarding any of the attitude statements other than substantial agreement that e-cigarettes were addictive. We also found that although students reported high levels of confidence in discussing traditional cigarette use with patients, most students reported low levels of confidence in discussing e-cigarette use with patients. A clear majority of students noted inadequate education about e-cigarettes, and although upperclassmen reported more confidence discussing traditional cigarette use with patients than first year medical students, students in the last year of medical school were no more confident than incoming first years in discussing e-cigarettes with patients.

These results are consistent with the findings of a 2014 survey of New York medical students, who felt less confident providing alternative tobacco product cessation counseling with patients than traditional cigarette counseling, and were less likely to report actual counseling about alternative tobacco products than about cigarettes [9]. These findings suggest students are currently ill-equipped to discuss e-cigarette use with their patients. Additionally, over twice as many participants noted receiving information about e-cigarettes through social media than through education in medical school. Medical education and social media are of course not at all equivalent as sources of information, but it is important to note that participants are being exposed to information about e-cigarettes outside of the curriculum, leading to a strong argument that correct factual knowledge must be provided to students during medical school.

Our survey found that 14.7% of University of Minnesota medical students had ever tried e-cigarettes while the 2014 New York survey found that only 4.1% of participants had ever tried an e-cigarette [9]. The reason for the difference in incidence of e-cigarette use is unclear, but may be partially attributed to the rapid increase of e-cigarette use in recent years and the approximately 1.5 year difference and/or the geographic distance between the two surveys. This level of use is additionally surprising as other surveys have found ever use of e-cigarettes is lower in respondents with a college degree [15]. However, less than 1 % of respondents to our survey were current e-cigarette users, compared to 1.6% of New York University medical students [9]. Both medical school surveys reported lower current use than an estimated 3.7% of adults in the United States identifying as current users [7].

We found no statistically significant differences in e-cigarette use by race or ethnicity. However, due to the high numbers of white students and small numbers in several racial categories, we may have been unable to detect actual differences between groups. We found a gender difference in that those who had ever tried e-cigarettes were predominantly male. Prior surveys have shown mixed results with some demonstrating higher prevalence of e-cigarette ever use in males [7, 16], while others show higher prevalence in females [2]. In our study, both male gender and ever use of e-cigarettes were associated with the attitudes that e-cigarettes should be recommended for smoking cessation, are better for patients’ health than other tobacco products, and have a reduced risk of cancer compared to traditional cigarettes. These students also expressed more confidence discussing e-cigarettes with patients. However, there is significant overlap between the male students and e-cigarette ever users with potential for confounding. Students who have previously tried e-cigarettes may also feel more confident because they are discussing a product they have personally experienced.

A survey of primary care physicians in the U.S. (*n* = 288) found that physicians who recommended use of e-cigarettes for smoking cessation were more likely to be male and have higher confidence in their own e-cigarette counseling skills [17],suggesting personal confidence and gender could play a role in physician behaviors regarding e-cigarettes. A separate analysis from this same dataset found knowledge scores (assessed via 5 questions) had an effect on whether a primary care physician intended to recommended e-cigarettes to their patients [18]. This suggests physician knowledge about e-cigarettes may influence physician behaviors, although these studies had limited generalizability due to a low response rate of 29%.

A notable strength of the study is the relatively high response rate (66.9%) in a large medical school. This might have been influenced by having a 4th year medical student as principal investigator, the use of social media to distribute the survey, and the $5 incentive. Another strength of this study is that it explores U.S. medical student attitudes and knowledge about e-cigarettes in greater depth than previously reported. Additionally, this is the first study to examine whether experience with e-cigarettes or other demographic variables are associated with differences in knowledge or attitudes about e-cigarettes.

There are a few limitations to this study. We deliberately did not include questions regarding traditional cigarette, alternative tobacco, or illicit drug use to have a brief and focused study on e-cigarettes, however this information would likely have been informative and interesting to compare with our e-cigarette data. Another limitation is that self-reported responses regarding the adequacy of the medical school’s curriculum are subject to recall bias. Additionally, the findings of this study are less generalizable since they represent responses from just one medical school’s students. Potential for non-response bias exists since participants were not required to answer every question, however there was a 97.7% survey completion rate among participants (*n* = 643/658).

Further research could survey other medical schools, graduate medical education and additional health team learners (e.g. pharmacy, dental, nursing students). It would also be prudent to assess if there is an association between medical student attitudes about e-cigarettes and confidence levels in discussing e-cigarettes with patients.

As prevalence of e-cigarette use has increased in recent years [1, 2], so have questions to providers from patients regarding e-cigarettes [19]. Studies have shown variability in attitudes toward e-cigarettes amongst providers [10, 12, 19] and varying advice is given [11, 20]. This is an issue that needs to be addressed at the undergraduate medical education level. Although multiple professional medical associations have developed guidelines about e-cigarettes [3–5, 21], it is not clear that this information is being disseminated to medical students. This study provides evidence that further curriculum development regarding e-cigarettes is urgently needed. We recommend that medical schools incorporate education about e-cigarettes into their curriculum, and that further research focuses on developing an encompassing tobacco/nicotine curriculum that includes e-cigarettes so that future physicians will be adequately prepared to confidently discuss all forms of nicotine products with their patients.

## Conclusions

In conclusion, this survey provides important information regarding medical students’ knowledge and experience regarding e-cigarettes, and it identifies gaps in medical school education at this school. As prevalence of e-cigarette use is likely to continue to increase, it is imperative that medical students receive more education about this important public health issue.

## Additional file

Additional file 1: Survey Questions. Additional file is a complete list of survey questions and responses answered by participants. (DOCX 15 kb)

## Abbreviations

e-cigarette: Electronic cigarette
