# Supplementary material for: What do medical students know about e-cigarettes? A cross-sectional survey from one U.S. medical school
Source: BMC Med Educ. 2018 Mar 2;18:32. doi: 10.1186/s12909-018-1134-1 (PMC5834872; doi:10.1186/s12909-018-1134-1)
Supplement: Supplementary file 1 — Survey Questions. Additional file is a complete list of survey questions and responses answered by participants. (DOCX 15 kb) [file 12909_2018_1134_MOESM1_ESM.docx]

Additional File 1: Survey Questions

| **Question** | **Possible responses** |
| --- | --- |
| **1) Experience** |  |
| Have you ever personally tried an e-cigarette? | Yes/No |
| If you have used e-cigarettes in the past, on average how frequently were you using them? | Less than once per month/monthly/weekly/daily |
| Do you currently use e-cigarettes? | Yes/No |
| How frequently do you currently use e-cigarettes? | Less than once per month/monthly/weekly/daily |
| How many times per day do you usually use your e-cigarette? (Assume that one “time” consists of around 15 puffs or lasts around 10 minutes) | Write-in |
| How soon after you wake up do you first use your e-cigarette? | Write-in |
| Do any of your immediate family members or close friends use e-cigarettes? | Yes/No |
| **2) Knowledge and Attitudes** |  |
| -Are e-cigarettes approved by the FDA for smoking cessation? | Yes/No/Not Sure |
| -Do you believe that e-cigarettes lower the risk of cancer for patients who use them instead of smoking traditional cigarettes? |  |
| -Do you believe e-cigarettes are a helpful aid for smoking cessation? |  |
| -If you were to see a patient who smokes cigarettes today, would you recommend the use of e-cigarettes as a smoking cessation method? |  |
| -Despite the unknowns, the use of e-cigarettes is better for my patients than smoking tobacco products. | Strongly Agree/Agree/Neither Agree  or Disagree/Disagree/Strongly Disagree |
| -E-cigarettes are addictive. |  |
| -It is important for physicians to be educated about e-cigarettes. |  |
| -As a student, I feel confident about my ability to discuss traditional cigarette use with my patients. |  |
| -As a student, I feel confident about my ability to discuss e-cigarette use with my patients |  |
| **3) Education** |  |
| Have you received any education about e-cigarettes in medical school? | Yes/No |
| Where did you learn about e-cigarettes? Check all that apply. (If “required”, “elective”, or “other” was selected, participants were given a blank space to fill in which clerkship or to please describe other). | Year 1 or 2 required curriculum, Year 3 or 4 required clerkship, Year 3 or 4 elective clerkship, Student interest group or optional lunch lecture, Grand Rounds, Informal interaction with MD/team, Other |
| In your opinion, have you received adequate education about e-cigarettes in medical school? | Yes/No |
| Where do you think the best time/location is to receive education about e-cigarettes in medical school? (If “required”, “elective”, or “other” was selected, participants were given a blank space to fill in which clerkship or to please describe other). | Year 1 or 2 required curriculum, Year 3 or 4 required clerkship, Year 3 or 4 elective clerkship, Student interest group or optional lunch lecture, Grand Rounds, Informal interaction with MD/team, Other |
| Have you received any information about e-cigarettes outside of medical school? Check all that apply. (If “other”, participants were asked to please describe) | Social media (Facebook, Twitter), Online advertising, Television advertisement, Radio advertisement, Billboards and/or public signs, Newspapers or Magazines, Other |
